# Phosphate addition intensifies the increase in N_2_O emission under nitrogen deposition in wet meadows of the Qinghai-Tibet Plateau

**DOI:** 10.3389/fmicb.2024.1472792

**Published:** 2024-12-18

**Authors:** Jiangqi Wu, Haiyan Wang, Guang Li

**Affiliations:** ^1^College of Forestry, Gansu Agricultural University, Lanzhou, China; ^2^College of Urban Environment, Lanzhou City University, Lanzhou, China

**Keywords:** nitrogen addition, phosphate addition, N_2_O flux, *NirK* gene, alpine wet meadow, Qinghai-Tibet Plateau

## Abstract

Alpine wet meadows are known as N_2_O sinks due to nitrogen (N) limitation. However, phosphate addition and N deposition can modulate this limitation, and little is known about their combinative effects on N_2_O emission from the Qinghai-Tibet Plateau in wet meadows. This study used natural wet meadow as the control treatment (CK) and conducted experiments with N (CON_2_H_4_ addition, N15), P (NaH_2_PO_4_ addition, P15), and their combinations (CON_2_H_4_ and NaH_2_PO_4_ addition, N15P15) to investigate how N and P supplementation affected soil N_2_O emissions in wet meadow of QTP. Contrary to previous studies on grasslands, the effect of phosphate addition treatment on soil N_2_O flux was not detectable during the growing seasons of 2019 and 2020. Over a span of two years, the N addition treatment significantly increased the N_2_O flux by 3.45 μg⋅m^–2^⋅h^–1^ due to increased soil N availability. Noticeably, phosphate addition intensified the effect of N deposition treatment on soil N_2_O flux with high significance in the early growth season of 2020. This augmentation can be attributed to the alleviation of limiting factors imposed by plants and microorganisms on soil N and P, fostering the mineralization and decomposition of litter and soil nutrients by microorganisms. Consequently, the results showed that total nitrogen and nitrate nitrogen were the main controls on soil N_2_O emission under N and P addition. In addition, redundancy analysis showed that the relative abundance of *NirK* genes in soil microorganisms (*Bradyrhizobium*, *Devosia*, *Ochrobactrum*, *Alcaligenes*, *Rhizobium*) is the main factor affecting N_2_O flux and available nitrogen. We project that if nutrient input continues to increase, the main limiting factor of soil will change from N restriction to P restriction due to the unique microbial nitrogen conversion process in the alpine meadow, significantly increasing N_2_O emissions. Consequently, the heightened contribution of alpine wet meadows to global warming and ozone depletion hinges on the dynamics of nutrient input regimes, spotlighting the urgent need for informed environmental management strategies.

## 1 Introduction

Nitrous oxide (N_2_O) is a significant driver of global warming, with a warming potential 298 times that of carbon dioxide per unit mass (Wu et al., 2023). Recent studies underscore the alarming reality that atmospheric N_2_O accounts for approximately 6.2% of anthropogenic global warming and is on a trajectory of annual increase at a rate of 0.2–0.3% (Kim et al., 2022). This potent greenhouse gas not only contributes to global warming but also poses a risk to the ozone layer, amplifying the impact of ultraviolet radiation and imperiling the health and survival of both human beings and other organisms. In wetlands characterized by abundant organic matter and surface water, N_2_O production predominantly occurs through denitrification processes (Mehnaz and Dijkstra, 2016; Martínez-Espinosa et al., 2021). Recognized as a crucial natural source of N_2_O, wetland soil denitrification significantly influences the global nitrogen biogeochemical cycle (Xie et al., 2015). Studies have shown that nutrient addition enhances the diversity and complexity of soil bacteria, especially altering the functional bacteria related to soil nutrient cycling (Wen et al., 2022; Zhao et al., 2023). Consequently, the inhibition of the denitrification process in wetland soils emerges as a key strategy for curbing N_2_O emissions, thereby mitigating environmental pollution risks and enhancing nitrogen use efficiency (Bardon et al., 2017).

Nitrogen and phosphorus are the key nutrient factors that limit plant growth in wetland ecosystems, and play a very important role in wetland productivity and soil ecological process (Gong and Wu, 2021). In recent years, nitrogen and phosphorus inputs to global ecosystems have increased dramatically due to excessive use of chemical nitrogen fertilizers and human overgrazing. N can be input into the wetland ecosystem through natural settlement and plant N fixation, while P is mostly input through livestock manure and artificial fertilization (Jiang et al., 2013). The high imbalance of N and P inputs in the 20th century (Peñuelas and Sardans, 2022), which is more pronounced in areas of high N settlement (Sun et al., 2017), may lead to a shift from N constraint to a broader P constraint or NP co-constraint (Wu et al., 2023). Studies have shown that nitrogen and phosphorus input can disrupt the nutrient balance of wetland ecosystems, which may affect soil nutrient status, soil microbial diversity and vegetation productivity in the ecosystem (Poeplau et al., 2019), and these can promote (Kuypers et al., 2018) or inhibit (Mori et al., 2014) N_2_O emissions in wetlands. Although the P input can alleviate the P limit of the system and the imbalance between N and P, it also affects the denitrification of wetland soil N (Kim et al., 2015) and increases N_2_O emissions (Wang et al., 2017). In addition, P input can reduce the P limitation of plants and soil microorganisms, promote nitrogen absorption and fixation of plants and microorganisms, and reduce the nitrogen substrate available for N_2_O production, thus inhibiting the emission of N_2_O from the system (Yu et al., 2017). Furthermore, P input can alleviate the P-limiting effect of denitrifying bacteria, promote bacterial activity (DeForest and Otuya, 2020), increase the abundance of functional genes produced by N_2_O, and promote N_2_O emission (Zhang et al., 2019). Therefore, an accurate understanding of the N_2_O emission characteristics and driving mechanism under the background of future N and P input is a vital prerequisite for slowing down wetland N_2_O emissions and coping with future climate change.

The Qinghai-Tibet Plateau (QTP) is a crucial ecological security barrier in China. It is home to a distinctive alpine meadow ecosystem, covering 53% of its total area (Zhou et al., 2020). Perennial low temperatures limit N and P mineralization in this area, configuring a nutrient limitation model marked by phosphorus or combined nitrogen and phosphorus limitation (Chen et al., 2018; Wu et al., 2020). Over time, global nitrogen deposition and large-scale overfertilization have propelled a surge in nitrogen and phosphorus deposition on the Qinghai-Tibet Plateau, escalating from 1 kg N ha^–1^ y^–1^ in 1980 to 5 kg N ha^–1^ y^–1^ in 2010, and projections indicating a twofold to threefold increase in the future (Liu et al., 2013). In addition, QTP is an essential base for developing animal husbandry in China, which produces a large amount of livestock manure and increases the input of phosphorus in wetland soil (Zhang et al., 2019). N deposition and increased P input not only affect the N and P content of wetland soil but also influence its physical and chemical properties and relative abundance of microbial dominant genera (Qiu et al., 2023; Voigt et al., 2020; Gao et al., 2019), which in turn impacts wetland N_2_O emissions. N addition can induce changes in soil N and P availability, influencing the structure and relative abundance of soil microbial communities, resulting in positive (Wu et al., 2023) or negative (Gao et al., 2014) effects on N_2_O flux. Conversely, the impact of increased phosphorus input on wetland N_2_O emissions varies, with some studies suggesting a promotion and others noting no significant effect (Gao et al., 2015; Zhang et al., 2019). The high water content and rich organic matter characteristic of the QTP area create a low-oxygen environment in high-altitude soils, fostering N_2_O production primarily through denitrification (Wrage-Mönnig et al., 2018). Central to this process is the *NirK* gene, acknowledged as the pivotal gene in denitrification, exceeding the significance of the *nirS* gene in soil denitrification processes (Wu et al., 2022). The presence and activity of the *NirK* gene directly influences the soil nitrogen cycle, thereby shaping the dynamics of soil N_2_O flux. In addition, soil water content and temperature are major factors affecting the diversity of denitrifying bacteria by regulating soil nitrogen content and oxygen content (Na et al., 2019). Therefore, it was crucial to investigate how soil nutrients and *NirK* genes respond to changes in N and P addition for a clearer understanding of N_2_O dynamics.

Therefore, this study focuses on the Gahai wet meadow as the research area in which to study the effects of N and P addition on N_2_O flux, soil nitrogen components, and denitrification functional genes. Our research objectives are threefold: (1) To determine how soil N_2_O flux responds to N and P addition, and whether this relationship changes with temperature and soil water content; (2) To clarify the relationship between the changes in surface soil nitrogen components and denitrification functional genes after the N and P addition; (3) To explore the effects of N and P addition on N_2_O flux through key abiotic and biological factors. We hypothesize that: (1) phosphate addition increases N_2_O emissions due to stimulating the activity of denitrifying microorganisms (Cui et al., 2018); (2) Nitrogen deposition promotes soil N_2_O emissions due to the lifting of nitrogen restrictions, and phosphate addition intensifies the impact of N deposition on soil N_2_O release; (3) N and P addition can change the soil N_2_O emissions by changing the relative abundance of dominant species in the soil denitrification bacterial community; (4) Soil nitrogen components, temperature, and water content are the main environmental controls for N_2_O emissions.

## 2 Material and methods

### 2.1 Study area

The research area was located in the nature reserve of Gahai-Zecha (33° 58′ 12″−34° 32′ 16″ N, 102° 05′ 00″−102° 47′ 39″ E), near the northeast border of the QTP. The distribution area of alpine meadows in the area was over 80%, with an average elevation of 3,430–4,300 m and an area of 57,846 hm^2^. The Gahai Wetland belongs to the highly cold and humid climate zone of the Qinghai-Tibet Plateau. The average temperature in the area from 1981 to 2020 was 2.9°C, with an average annual precipitation of 785 mm.^1^ More than 70% of the rainfall was concentrated during the growing season (Wang et al., 2022). The soil was mainly composed of meadow soil, peat soil, and swamp soil, with a high organic matter content.

### 2.2 Experimental design

In May 2019, we selected an area characterized by flat terrain and evenly distributed vegetation as the experimental area. To implement nitrogen and phosphorus treatments, we utilized urea (CON_2_H_4_) and sodium dihydrogen phosphate (NaH_2_PO_4_) to convert these substances into their respective nitrogen and phosphorus content (Camenzind et al., 2014). Studies have shown that the current background value for both nitrogen deposition and phosphorus addition in this area was 5.0 kg N ha^–1^ y^–1^, and the rate of nitrogen deposition and phosphorus addition is expected to increase 2–3 times in the future (Zhai et al., 2024; Wu et al., 2023). Based on this, we added 15 kg N ha^–1^ y^–1^ and 15 kg P ha^–1^ y^–1^ as nutrient additions under different treatments. A total of four treatments were set up, including no fertilization as the control (CK), N15 treatment (CON_2_H_4_ addition), P15 treatment (NaH_2_PO_4_ addition), and N15P15 treatment (CON_2_H_4_ and NaH_2_PO_4_ addition). Each treatment had three replicates, a total of 12 plots. The plot size was 2 m × 2 m, and to avoid mutual interference, the distance between two adjacent plots was 5 m. At the end of May 2019 and 2020, the corresponding mass of fertilizer was dissolved in 2 L of water, and the sprayer was used to uniformly distribute the fertilizer solution across the experimental plots, while the control plots received an even spray of 2 L of water.

### 2.3 N_2_O flux and soil sample collection

Throughout the plant growth season, spanning from June to October 2019 and 2020, we conducted comprehensive measurements of N_2_O fluxes for each treatment. Gas samples were meticulously collected at 15-day intervals, employing the closed static chamber technique (Ma et al., 2018). The chambers used in our study were designed with dimensions of 50 cm in length, width, and height. After chamber closure, gas samples were taken at specific time intervals (0, 3, 10, 25, and 40 min) after chamber closure. Following collection, the N_2_O gas samples of the air samples were quantified using a gas chromatograph (Echrom A90, China). For N_2_O flux, the concentration slope versus time was accepted if *R*^2^ > 0.80 (Schneider et al., 2023). The N_2_O fluxes were then calculated using linear regression of N_2_O in the chamber concentrations versus time (Shi et al., 2021). Overall, 99.5% of the fluxes were calculated by linear regression and 0.5% by non-linear regression, and all N_2_O flux data were used for further analysis. In addition, a portable digital thermometer (JM624) and a soil moisture content analyzer (RS232) were used to measure soil temperature (ST) and soil water content (SWC) at 10 cm depth.

We collected 0–10 cm soil samples from June to October 2019 and 2020 (mid-month; June is the early growth period, EG; July to August is the middle growth period, MG; September to October is the late growth period, LG). The soil characteristics, including soil ammonium nitrogen (NH_4_^+^), nitrate nitrogen (NO_3_^–^), total nitrogen (TN), total phosphorus (TP), microbial biomass nitrogen (MBN), and microbial biomass carbon (MBC) were determined using previously established methods described (Saha et al., 2018; Wu et al., 2023). Additionally, on 20 June, 1 August, and 22 September 2020, liquid nitrogen was used to transport 0–10 cm soil samples back to the laboratory for testing the *NirK* gene sequence in each treated soil using a detection method as described in previous research (Zhang et al., 2018; Wu et al., 2022). DNA was extracted from 0.25 g of soil samples using the soil DNA extraction kit (MoBio Laboratories, Carlsbad, CA, USA), and use 1% agarose gel electrophoresis solution was used to detect DNA quality. The eligible DNA genes were amplified using the primer combination nirK1F-nirK5R (Chen et al., 2010), and the amplified sequencing was performed using the Illumina MiSeq 250 Sequencer (generating 2 × 250 bp paired-end reads) from Shanghai Tianhao Biotechnology Co., Ltd.

### 2.4 Statistical analysis

A normality test was conducted on the data using SPSS 22.0 software. The effects of treatments on soil N_2_O emissions and soil characteristic variables (ST, SWC, NH_4_^+^, NO_3_^–^, TP, MBC, and MBN) were tested with a one-way ANOVA. A linear regression model was used to explain the variability of environmental variables to N_2_O flux. Additionally, the differences in the *NirK* gene community structure were displayed by principal coordinate analysis (PCoA) based on Bray-Curtis distances using the PCoA function in the “ape” package in R software (significance level was *P* < 0.05). Redundancy analysis (RDA) was used to explore the relationships between soil *NirK* gene community structure, N_2_O fluxes, and soil characteristic factors.

## 3 Results

### 3.1 Soil characteristics

**FIGURE 1:**
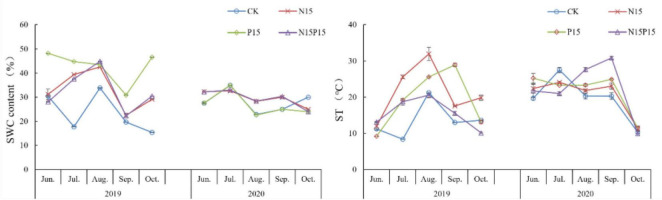
Changes in soil water content and soil temperature under nitrogen and phosphate addition. Error bars show the standard error. CK, Control; N15, CON_2_H_4_ addition treatment; P15, NaH_2_PO_4_ addition treatment; N15P15, CON_2_H_4_ and NaH_2_PO_4_ addition treatment.

The soil characteristics of the 0–10 cm soil layer in the wet meadow on the QTP were significantly affected by the N and P addition treatments (Table 1 and Figure 1). Compared with CK, P15 and N15P15 treatments significantly increased the contents of soil MBN, NH_4_^+^-N, NO_3_^–^-N, and MBC (*P* < 0.05); P15 treatments significantly increased the soil TP content (*P* < 0.05), while N15 treatments significantly decreased the contents of TN, TP, NO_3_^–^-N and MBC (*P* < 0.05). ST showed a trend of increasing first and then decreasing after a month, while there was no clear SWC trend. In addition, the soil characteristics content under four treatments showed significant seasonal variations (*P* < 0.05, Table 2). Except for the TP content in the soil treated with CK and N15, the content of MBN, TN, NH_4_^+^, and NO_3_^–^ in the soil showed a trend of first increasing and then decreasing with the extension of the season, with the larger values appearing in MG. The soil MBC content under the four treatments showed a trend of decreasing and increasing with the extension of seasons. Repeated analysis of variance demonstrated that N and P addition and season significantly interaction influenced the soil characteristics content in wet meadow soil (Table 2).

**TABLE 1:** Changes in surface soil characteristics during different vegetation growth seasons under nitrogen and phosphate addition for two consecutive years (mean ± standard errors).

		MBN (abs⋅g^–1^ dry soil)	TN (g⋅kg^–1^)	TP (mg⋅kg^–1^)	NH_4_^+^-N (mg⋅kg^–1^)	NO_3_^–^-N (mg⋅kg^–1^)	MBC (mg⋅kg^–1^)
EG	CK	0.13 ± 0.010 b	5.79 ± 0.20 b	71.08 ± 2.49 a	39.23 ± 0.37 b	26.67 ± 0.61 b	1,129.74 ± 68.95 b
	N15	0.17 ± 0.008 a	4.99 ± 0.35 c	60.60 ± 0.95 b	38.75 ± 0.71 b	18.82 ± 0.55 c	1348.87 ± 42.80 ab
	P15	0.20 ± 0.004 a	6.93 ± 0.06 a	36.14 ± 2.57 c	51.75 ± 1.66 a	35.86 ± 1.06 a	1,534.77 ± 75.48 a
	N15P15	0.11 ± 0.010 b	6.10 ± 0.15 b	40.52 ± 0.01 c	40.22 ± 1.63 b	24.97 ± 0.80 b	1,320.72 ± 26.66 ab
MG	CK	0.17 ± 0.005 c	6.30 ± 0.10 a	48.23 ± 5.57 b	44.61 ± 2.27 c	43.17 ± 2.24 c	693.40 ± 23.71 c
	N15	0.17 ± 0.006 c	5.66 ± 0.09 b	50.98 ± 4.82 b	76.54 ± 3.21 b	42.68 ± 2.67 c	740.46 ± 20.09 c
	P15	0.24 ± 0.003 b	5.61 ± 0.11 b	104.25 ± 5.91 a	85.09 ± 1.04 a	57.81 ± 1.94 a	936.82 ± 13.20 b
	N15P15	0.29 ± 0.006 a	6.73 ± 0.25 a	89.11 ± 0.52 a	89.77 ± 0.41 a	49.85 ± 0.43 b	1,150.44 ± 10.25 a
LG	CK	0.10 ± 0.009 b	3.26 ± 0.09 b	84.67 ± 1.89 a	45.72 ± 0.54 b	35.30 ± 0.63 c	1,522.85 ± 24.81 b
	N15	0.11 ± 0.002 a	3.66 ± 0.11 a	46.16 ± 0.73 b	33.42 ± 0.21 c	33.55 ± 1.87 c	1,153.56 ± 40.66 c
	P15	0.11 ± 0.002 a	2.78 ± 0.16 c	85.11 ± 1.92 a	44.06 ± 0.89 b	42.01 ± 1.19 b	1,518.24 ± 56.76 b
	N15P15	0.08 ± 0.001 c	3.71 ± 0.10 a	47.21 ± 1.62 b	84.60 ± 0.10 a	48.45 ± 1.94 a	2,404.55 ± 12.39 a
Average	CK	0.13 ± 0.005 c	5.12 ± 0.07 b	67.99 ± 1.66 b	43.19 ± 0.79 d	35.05 ± 1.05 c	1,115.33 ± 14.32 c
	N15	0.15 ± 0.005 b	4.77 ± 0.11 b	52.58 ± 1.68 d	49.57 ± 0.84 c	31.68 ± 1.30 d	1,080.96 ± 31.96 c
	P15	0.18 ± 0.001 a	5.10 ± 0.04 b	75.17 ± 0.47 a	60.30 ± 0.08 b	45.23 ± 0.58 a	1,329.94 ± 47.56 b
	N15P15	0.16 ± 0.003 b	5.51 ± 0.16 a	58.95 ± 0.37 c	71.53 ± 0.59 a	41.09 ± 1.04 b	1,625.24 ± 37.70 a

MBN, microbial biomass nitrogen; TN, total nitrogen; TP, total phosphorus; NH_4_^+^-N, ammonium nitrogen; NO_3_^–^-N, nitrate nitrogen; MBC, microbial biomass carbon. Different lowercase letters represent significant differences (*P* < 0.05) between the treatments.

**TABLE 2:** Results of a repeated-measures ANOVA testing for differences in surface soil characteristics (TN, NO_3_^–^, NH_4_^+^, MBN, DON) among nitrogen and phosphate addition using season as the repeated variable.

Source of variation	Df	MBN		TN		TP		NH_4_^+^		NO_3_^–^		MBC		N_2_O	
		**F**	***P***	**F**	***P***	**F**	***P***	**F**	***P***	**F**	***P***	**F**	***P***	**F**	***P***
T	3	39.944	0.000	9.974	0.000	31.269	0.000	233.784	0.000	47.918	0.000	73.911	0.000	35.689	0.000
S	2	388.236	0.000	342.137	0.000	48.238	0.000	525.697	0.000	209.990	0.000	235.054	0.000	86.075	0.000
T × S	6	56.581	0.000	15.207	0.000	65.598	0.000	123.369	0.000	7.851	0.000	32.596	0.000	8.241	0.000

T, treatment; S, season; MBN, microbial biomass nitrogen; TN, total nitrogen; TP, total phosphorus; NH_4_^+^-N, ammonium nitrogen; NO_3_^–^-N, nitrate nitrogen; MBC, microbial biomass carbon.

### 3.2 N and P addition effects on N_2_O flux

**FIGURE 2:**
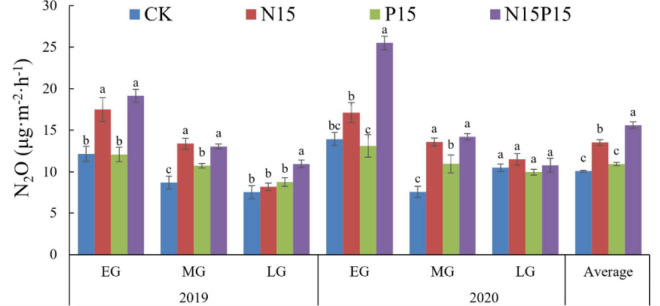
Average N_2_O flux in the 2019 and 2020 early (EG), middle (MG), and late (LG) growing season under four treatments. Average N_2_O flux in the 2019 and 2020 early (EG), middle (MG), and late (LG) growing season under four treatments. Different lowercase letters represent significant differences (*P* < 0.05) between the treatments, error bars show the standard error. CK, Control; N15, CON_2_H_4_ addition treatment; P15, NaH_2_PO_4_ addition treatment; N15P15, CON_2_H_4_ and NaH_2_PO_4_ addition treatment.

High levels of N addition and NP addition significantly impacted the 2-year average N_2_O flux, but P addition did not significantly affect N_2_O flux (Figure 2). Throughout the entire plant growing seasons (June to October) of QTP in 2019 and 2020, the N_2_O emissions varied between the four treatments. Compared with CK (10.08 μg⋅m^–2^⋅h^–1^), N15, P15, and N15P15 increased N_2_O average emission by 3.46, 0.86, and 5.53 μg⋅m^–2^⋅h^–1^ in wet meadow. Moreover, P addition increased the increase of N_2_O emission resulting from N fertilization by 2.07 μg⋅m^–2^⋅h^–1^ (Figure 2).

Fertilization and seasonal changes also significantly impacted soil N_2_O flux in QTP wet meadow (Table 2). Compared with the CK treatments in the EG (12.17 ± 0.90 μg⋅m^–2^⋅h^–1^) and MG periods (8.71 ± 0.79 μg⋅m^–2^⋅h^–1^) of 2019, the N15 treatment significantly increased average N_2_O emissions by 5.34 μg⋅m^–2^⋅h^–1^ during the EG period and 4.67 μg⋅m^–2^⋅h^–1^ during the MG period. Likewise, soil N_2_O emissions under N15 treatment increased by 3.16 μg⋅m^–2^⋅h^–1^ during the EG period and 5.99 μg⋅m^–2^⋅h^–1^ during the MG period in 2020. In addition, soil N_2_O emissions under the interactive treatment of N15 and P15 fertilization treatment were 6.95 μg⋅m^–2^⋅h^–1^ and 11.56 μg⋅m^–2^⋅h^–1^ higher than under the CK treatment in the EG of 2019 and 2020, respectively (Figure 2). Additionally, P fertilization together significantly increased the N_2_O flux by 2.02 and 3.37 μg⋅m^–2^⋅h^–1^ compared with the CK treatment only during the MG period in 2019 and 2020 (*P* = 0.03 and *P* = 0.01). In the late growth season, all other treatments had no significant effect on N_2_O flux except for N15P15 (*P* > 0.05).

### 3.3 Effect of N and P addition on soil *NirK* genes

**FIGURE 3:**
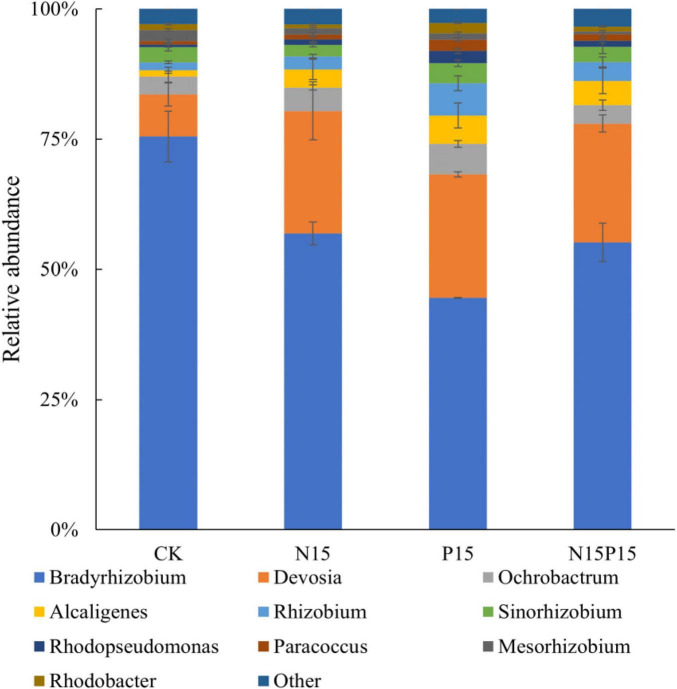
Histogram of soil *NirK* bacterial community structure (genus level) under nitrogen and phosphate addition treatment. CK, Control; N15, CON_2_H_4_ addition treatment; P15, NaH_2_PO_4_ addition treatment; N15P15, CON_2_H_4_ and NaH_2_PO_4_ addition treatment.

**FIGURE 4:**
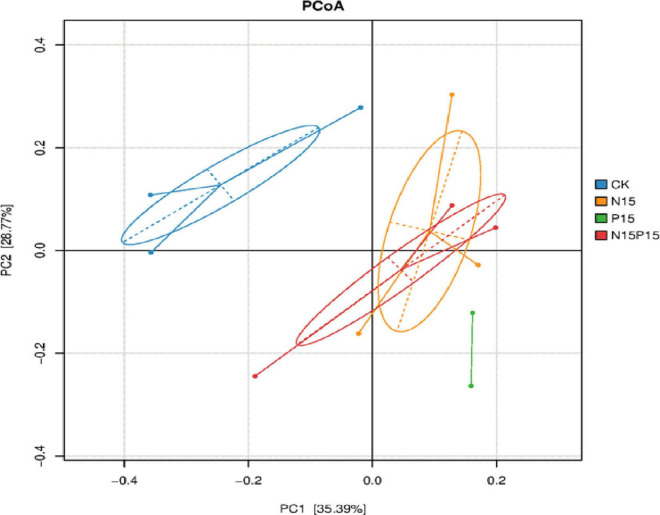
Principal coordinates analysis (PCoA) of bacterial community structure based on Bray-Curtis distances under different treatment. CK, Control; N15, CON_2_H_4_ addition treatment; P15, NaH_2_PO_4_ addition treatment; N15P15, CON_2_H_4_ and NaH_2_PO_4_ addition treatment. We used the first two samples from the P15 treatment for PCoA analysis due to the third soil sample from the P15 treatment was contaminated.

There were significant differences in the composition of soil microbial communities under the four N and P addition treatments (Figure 3). Principal coordinates analysis (PCoA) of Bray-Curtis distance showed that there were significant differences in soil microbial community structure between N and P addition treatments and CK (*P* = 0.024), while the differences in the microbial community structure between N15 and N15P15 treatments did not reach significant (*P* = 0.152). Moreover, the dominant genera among the four treatments in wet meadows were *Bradyrhizobium*, *Devosia*, *Ochrobactrum*, *Alcaligenes*, and *Rhizobium* (Figure 4). N and P addition (N15, P15, N15P15) significantly reduced the relative abundance of *Bradyrhizobium*, and increased the relative abundance of *Devosia*, *Ochrobactrum*, *Alcaligenes*, and *Rhizobium*.

### 3.4 Relationships between N_2_O flux and soil characteristics and *NirK* genes

**FIGURE 5:**
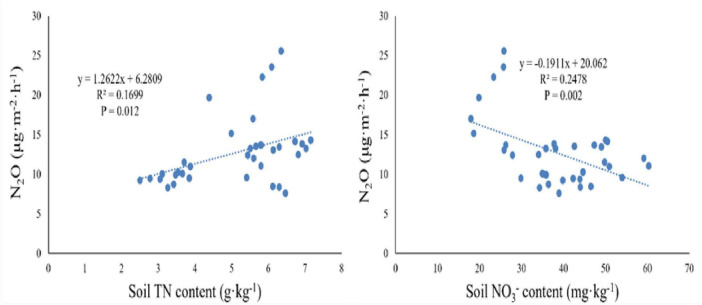
Relationship between soil N_2_O flux and two environmental variables (total nitrogen (TN) and nitrate nitrogen (NO_3_^–^) at 10 cm soil depths).

**FIGURE 6:**
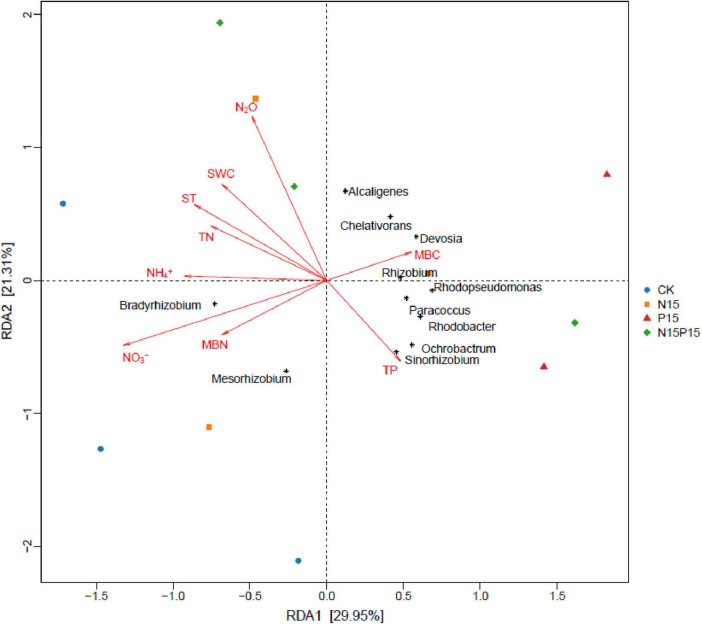
Redundancy analysis (RDA) between N_2_O emissions and NirK bacterial community structure (genus level) and soil characteristics. The length and angle of the arrows indicates the magnitude and direction of the correlation, respectively. CK, Control; N15, CON_2_H_4_ addition treatment; P15, NaH_2_PO_4_ addition treatment; N15P15, CON_2_H_4_ and NaH_2_PO_4_ addition treatment.

After two years of nitrogen and phosphorus addition treatments, significant correlations between N_2_O flux and TN at 10 cm depth, and between N_2_O flux and NO_3_^–^ at 10 cm depth were observed (Table 3), explaining 16.99 and 24.78% of N_2_O variation, respectively (Figure 5). Furthermore, the relationships between N_2_O flux and other soil characteristics (ST, SWC, MBN, TP, NH_4_^+^ and MBC at 10 cm depth) were undetectable in this study. Moreover, at the level of dominant genera, the N_2_O flux was significantly negatively correlated with the relative abundance of *Ochrobactrum*, significantly positively correlated with the relative abundance of *Alcaligenes* (*P* < 0.05, Figure 6), and weakly correlated with other dominant genera (*P* > 0.05). *Bradyrhizobium* was significantly positively correlated with NH_4_^+^, NO_3_^–^, MBN, and significantly negatively correlated with MBC. *Devosia* and *Rhizobium* were significantly positively correlated with MBC, while negatively correlated with NH_4_^+^, NO_3_^–^, and MBN. *Ochrobactrum* was positively correlated with TP content but negatively correlated with SWC, ST, and TN.

**TABLE 3:** Correlation analysis between soil characteristics and N_2_O flux in different vegetation growth seasons.

Parameter	SWC	ST	MBN	TN	TP	NH_4_^+^	NO_3_^–^	MBC
Df	36	36	36	36	36	36	36	36
F	0.277	−0.045	0.058	0.412	−0.306	−0.103	−0.498	0.226
*P*-value	0.102	0.793	0.736	0.012	0.070	0.552	0.002	0.129

SWC, soil water content; ST, soil temperature; MBN, microbial biomass nitrogen; TN, total nitrogen; TP, total phosphorus; NH_4_^+^-N, ammonium nitrogen; NO_3_^–^-N, nitrate nitrogen; MBC, microbial biomass carbon.

## 4 Discussion

### 4.1 Effects of nitrogen and phosphorus addition on N_2_O flux in wet meadow

N_2_O is mainly produced by two biological processes, nitrification and denitrification, which are affected by the soil environment and substrate nutrient content (Cui et al., 2016). After two years of phosphate addition treatment alone in the Qinghai-Tibet Plateau wet meadow, no significant effects on N_2_O fluxes were found. This fails to confirm our first hypothesis but is consistent with previous studies on alpine grasslands and wetlands (Wang et al., 2018; Zhang et al., 2019). The significant positive effect of phosphate addition on N_2_O flux has been reported in grasslands. It is attributed to the increase in soil-denitrifying microbes biomass and activity under the phosphate addition treatment (Cui et al., 2018). Although soil total phosphorus content was increased due to the phosphate addition treatment, no effect on N_2_O fluxes has been detected in this study. Contrary to the phosphate addition treatment, N fertilization (N15) significantly increased N_2_O emission, which confirms our second hypothesis that N fertilization relieves N element limitation in wet meadow systems and promotes N_2_O emissions. On the one hand, N and P addition treatment can increase soil available nitrogen (Table 1), promote the abundance of soil microbial functional genes, and increase the reaction matrix available for N_2_O production (Mori et al., 2017). On the other hand, nitrogen addition will weaken the limiting effect of nutrient elements, accelerate the decomposition rate of litter, increase the content and distribution of soil nutrients (Jiang et al., 2021), and promote the emission of N_2_O.

Similar to the second hypothesis, we found that phosphate addition trended to intensify the effect of N deposition treatment (N15P15) and it was significant in the early growth season of 2020 (Figure 2). The comprehensive impact of nitrogen and phosphorus addition on N_2_O emissions is mainly due to the competition between plants and soil microorganisms for nutrient availability (Shen and Zhu, 2022). The simultaneous addition of NP not only relieved the N limitation of the soil in the Gahai wet meadow, but also increased the available nutrients in the soil. Adequate P element increased the activity of soil microorganisms and extracellular enzymes (Mehnaz et al., 2019; Camenzind et al., 2016), promoted the mineralization of nitrogen in the ecosystem (Anderson et al., 2023), and stimulated the occurrence of denitrification (Gao et al., 2015), resulting in that the addition of P enhanced the influence of nitrogen deposition on N_2_O emission. To survive, soil microorganisms will promote the mineralization and decomposition of soil TN into available nitrogen (MBN, NH_4_^+^, NO_3_^–^), resulting in a lower N_2_O flux under phosphorus addition conditions than under nitrogen addition treatment. This result is consistent with Baral et al.’s (2014) finding that phosphorus addition reduces N_2_O emissions, mainly due to the Gahai wet meadow being an N limited ecosystem (Wu et al., 2021). Phosphorus addition reduces the absorption of soil mineral nitrogen by plant roots and nitrogen assimilation by soil microorganisms (Guan et al., 2024; Chen et al., 2023), ultimately reducing N_2_O emissions. Additionally, the N_2_O flux under N and P addition gradually decreased with the extension of the season (Figure 3), with the minimum value appearing at the end of plant growth (September-October), which was mainly related to the rapid depletion of nutrients after N and P addition (Gebremichael et al., 2022; Wei et al., 2020). On the one hand, the available nutrients in the soil gradually decrease with the extension of nitrogen and phosphorus addition time, and the nitrification and denitrification processes gradually weaken due to the decrease in substrate concentration, resulting in the peak of N_2_O flux at the beginning of plant growth after nitrogen and phosphorus addition. On the other hand, this temporal trend is linked to the rainfall pattern characteristic of the Gahai wet meadow area (Wu et al., 2023), with rainfall mainly concentrated in the plant growing season (May-October). The elevated temperatures during this period enhance soil microbial activity, fostering the microbial decomposition of nitrogen and phosphorus nutrients and litter. This, in turn, supplements the substrate concentration for soil microbial nitrification and denitrification processes (Luo et al., 2020), leading to an initial increase in N_2_O flux. However, as the growing season progresses, nutrient competition between plants and soil microorganisms emerges (Jones et al., 2018). Consequently, soil nutrient content and soil N_2_O flux gradually decline. In contrast, the N_2_O flux under the CK treatment displayed a trend of first decreasing and then increasing with the extension of the season. Because plant growth absorbs the available nutrients in the soil, the competition between soil microorganisms and plants for soil nutrients is intensified (Das et al., 2022). In addition, P addition alone significantly increased N_2_O emissions in the middle growing season, because higher temperatures increased N mineralization and decomposition by soil microorganisms and increased nutrient content in the soil (Table 1), P addition promoted soil microbial and extracellular enzyme activities that enhanced soil denitrification (Mori, 2022; Mehnaz et al., 2019; Baral et al., 2014), and contributed to N_2_O emissions. During the late growth period, aboveground plants will turn yellow or even die; the decomposition of aboveground litter by soil microorganisms increases the effective nutrients in the soil (Ochoa-Hueso et al., 2020), promotes soil nutrient cycling processes and increases the substrate content of N_2_O production (Pandeya et al., 2020), leads to an increase in N_2_O emissions under CK treatment at the late growth period.

### 4.2 Effects of environmental and microorganism factors on N_2_O flux

N and P addition not only induces shifts in the soil environmental factors of wet meadows (e.g., SWC and ST) but also leads to changes in soil nitrogen components (e.g., TN, NH_4_^+^, NO_3_^–^, MBN) and soil microbial community structure, ultimately changing soil N_2_O flux. Prior research has shown that soil total nitrogen and nitrate are substrates for nitrification and denitrification processes that produce N_2_O and soil microorganisms usually absorb mineral nitrogen for nitrification and denitrification, resulting in reduced nitrogen component content in the soil and promoting N_2_O emission (Kuang et al., 2018). Consistent with this understanding, our study reveals a significant correlation between soil N_2_O flux, nitrate nitrogen, and TN content (Table 2 and Figure 5). Moreover, prior research has consistently underscored soil temperature and humidity as principal drivers influencing N_2_O flux. This correlation stems from the direct impact of temperature on soil microbial activity, and the indirect influence of soil water content on denitrification processes by regulating anaerobic conditions (Bååth, 2018; Wu et al., 2022). Surprisingly, unlike the anticipated association outlined in our four hypotheses, no significant correlation was observed between N_2_O flux and temperature, NH_4_^+^, and MBN. This incongruity challenges the notion that soil temperature and nitrogen composition are the primary controllers of N_2_O flux. In contrast, our findings reveal a substantial positive correlation between N_2_O flux and SWC. This alignment emphasizes the influential role of soil water content in shaping the redox state and microbial activity within wet meadow soils. Increased SWC promotes denitrification processes, thereby influencing the production and transport of N_2_O (Xu et al., 2016).

Similar to the third hypothesis, we found that N addition and NP co-addition can promote soil N_2_O emissions by changing the relative abundance of dominant species in the soil denitrification bacterial community. Although P addition significantly altered the relative abundance of dominant species in the soil denitrification bacterial community, no significant effect was observed on the average N_2_O emission. Specifically, N and P addition resulted in a decrease in the relative abundance of *Bradyrhizobium* and an increase in the relative abundance of *Devosia* and *Rhizobium* genera. This observed microbial community restructuring aligns with prior research by Xu et al. (2019): as *Bradyrhizobium* is a slow-growing bacteria, N and P addition increases the available nitrogen content in the soil. Plants absorb a large amount of mineral nitrogen for root growth, which increases soil ventilation (Table 1). A higher concentration of oxygen will cause *Bradyrhizobium* to consume more energy to protect the nitrogenase from oxygen inactivation (Lin et al., 2018), decreasing the abundance and nitrogen component content of *Bradyrhizobium*. Consistent with previous studies showing that reduced microbial nitrogen fixation capacity increases N_2_O emissions (Mori et al., 2014), this confirms our second hypothesis that N and P addition alters N_2_O emissions by modifying the proportion of prevailing species within soil denitrifying bacterial communities. Moreover, as reflected in Table 1, the observed increase in the relative abundance of fast-growing nitrogen-fixation bacteria, such as *Devosia* and *Rhizobium*, due to nitrogen and phosphorus addition contributes to heightened soil microbial activity (MBN and MBC). This stimulation, in turn, facilitated the rapid propagation of *Devosia* and *Rhizobium*, directly promoting the soil nitrogen cycle (Kuypers et al., 2018; Fang et al., 2019). The consequence is an indirect increase in N_2_O emission. Notably, *Devosia* and *Rhizobium* are known to promote plant growth (Zhou et al., 2017; Yang et al., 2021). Consequently, the accelerated absorption of available nitrogen by wet meadow plants, facilitated by these bacteria, reduces soil nitrogen component content. This finding is consistent with the results demonstrating a negative correlation between *Devosia*, *Rhizobium*, and soil NH_4_^+^, NO_3_^–^, MBN, alongside a significant positive correlation with MBC (Figure 6). Contrastingly, *Ochrobactrum* exhibits a negative correlation with N_2_O, attributing to its capacity to degrade aromatic and hydrocarbons (Veeranagouda et al., 2006). This microbial activity converts high levels of NO_2_^–^ to N_2_ (Doi et al., 2009) through denitrification under anaerobic conditions. In this study, the addition of nitrogen and phosphorus resulted in higher soil available nutrient content, promoting the growth of soil microorganisms and increasing the relative abundance of *Ochrobactrum* (Figure 3). This, in turn, stimulated the conversion of NO_2_^–^ into N_2_, and reduced the production of N_2_O. Therefore, the availability of soil nitrogen and the relative abundance of microorganisms emerge as pivotal limiting factors for soil denitrification in QTP wet meadows. These intricate microbial and nutrient dynamics underscore the importance of considering alpine wet meadows in the prediction of global greenhouse gas emissions and climate models, particularly in relation to N_2_O emissions. The unique geography and sensitive climatic conditions of the Gahai wet meadow system have resulted in much higher N_2_O emissions than elsewhere (Tiemeyer et al., 2016; Jiang et al., 2010), soil microbes and effective nutrients are the main factors influencing N_2_O emissions. The N_2_O warming potential is about 296 times that of carbon dioxide, contributing about 7% to global warming (Feng and Li, 2023), becoming the main destroyer of the ozone layer. To accurately assess the contribution of global climate change and nutrient inputs to the warming effect, the model needs to clarify the response of soil N_2_O emissions to nutrient inputs (Prentice et al., 2012).

## 5 Conclusion

This study examined the effects of high levels of N and P additions on N_2_O flux within alpine wet meadow ecosystems. The impact of phosphate addition treatment on soil N_2_O flux was not detectable, while a significant effect of N deposition treatment was shown. A trend that phosphate addition intensified the effect of N deposition treatment on soil N_2_O flux was observed, which was significant in the early growth season. Among different abiotic factors, soil TN and NO_3_^–^ were the main controls for N_2_O emission, while SWC has a weaker impact on N_2_O flux. Furthermore, our research revealed a strong correlation between N_2_O flux and soil available nitrogen and the relative abundance of *NirK* microorganisms (*Bradyrhizobium*, *Devosia*, *Ochrobactrum*, *Alcaligenes*, *Rhizobium*). In the alpine wet meadow ecosystem, the denitrification process is constrained by nitrogen availability and microbial biomass carbon and phosphorus content. Our results indicated a shift in the main limiting factor from nitrogen to phosphorus in response to nutrient addition, suggesting a change in the ecological dynamics of this area. Nitrogen alone and nitrogen-phosphorus interactions were all found to significantly amplify the environmental pressure associated with N_2_O emission in wet meadows. This insight underscores the importance of considering the intricate interplay between nutrient dynamics, microbial communities, and environmental factors for a comprehensive understanding of the consequences of nutrient additions in alpine wet meadows.

## Data Availability

The original contributions presented in this study are included in this article, further inquiries can be directed to the corresponding author.

## Footnotes

1
http://data.cma.cn/data/weatherbk.html
